# Force Outputs during Squats Performed Using a Rotational Inertia Device under Stable versus Unstable Conditions with Different Loads

**DOI:** 10.1371/journal.pone.0154346

**Published:** 2016-04-25

**Authors:** Jairo Vázquez-Guerrero, Gerard Moras, Jennifer Baeza, Sergio Rodríguez-Jiménez

**Affiliations:** 1 Sport Performance, Institut Nacional d’Educació Física de Catalunya, Centre de Barcelona, Barcelona, Spain; 2 FCBarcelona, Barcelona, Spain; 3 Fundació Universitària del Bages, Manresa, Barcelona, Spain; University of Rome, ITALY

## Abstract

The purpose of the study was to compare the force outputs achieved during a squat exercise using a rotational inertia device in stable versus unstable conditions with different loads and in concentric and eccentric phases. Thirteen male athletes (mean ± SD: age 23.7 ± 3.0 years, height 1.80 ± 0.08 m, body mass 77.4 ± 7.9 kg) were assessed while squatting, performing one set of three repetitions with four different loads under stable and unstable conditions at maximum concentric effort. Overall, there were no significant differences between the stable and unstable conditions at each of the loads for any of the dependent variables. Mean force showed significant differences between some of the loads in stable and unstable conditions (*P* < 0.010) and peak force output differed between all loads for each condition (*P* < 0.045). Mean force outputs were greater in the concentric than in the eccentric phase under both conditions and with all loads (*P* < 0.001). There were no significant differences in peak force between concentric and eccentric phases at any load in either stable or unstable conditions. In conclusion, squatting with a rotational inertia device allowed the generation of similar force outputs under stable and unstable conditions at each of the four loads. The study also provides empirical evidence of the different force outputs achieved by adjusting load conditions on the rotational inertia device when performing squats, especially in the case of peak force. Concentric force outputs were significantly higher than eccentric outputs, except for peak force under both conditions. These findings support the use of the rotational inertia device to train the squatting exercise under unstable conditions for strength and conditioning trainers. The device could also be included in injury prevention programs for muscle lesions and ankle and knee joint injuries.

## Introduction

Traditional free-weight exercise is the most common form of resistance training, utilizing resistance provided by gravitational force. However, external load in resistance exercises can also be provided by using flywheel inertia resistance, which has become increasingly popular over the last two decades [1–8].

This method makes use of specialized devices that exploit the inertia momentum generated by a lightweight rotating flywheel as a source of resistance to the effort made by the trainee. The system differs from traditional free-weight forms of resistance exercise in that it generates resistance as a function of the mass, the distribution of the mass and the angular acceleration of the flywheel [2] during coupled concentric and eccentric actions [7]. This means that it offers gravity-independent resistance [2]. The inertia momentum of a rotating flywheel provides unlimited resistance throughout the entire range of any concentric action [1,7]. Moreover, brief episodes of eccentric overload may occur, and the peak value of force during flywheel inertia exercises is greater than during standard weight training [4,5]. The use of the flywheel resistance method suggests that training elicits early, robust neuromuscular adaptations [7].

To date, most authors who have studied the method have used YoYo^®^ devices (YoYo^®^ Technology AB, Stockholm, Sweden) and have focused their interest on closed- or open-chain single-joint exercises (i. e. leg extension, leg curl or leg press) [4,5,7,8], rather than on multi-joint movements like the squat exercise. However, other sorts of flywheel inertia resistance devices are available that differ slightly from YoYo^®^ devices, including the rotational inertia device (RID), which is used in the present research. The RID is a cone-shaped resistance exercise pulley machine that can be used through any range of motion and accommodates inertia momentum resistance at any speed [9].

The dynamic squat is an integral part of training programs for sports that require high levels of strength and power. It is an essential multiple-joint free-weight resistance exercise that primarily strengthens the ankle, knee and hip extensors and can improve athletic performance in skills such as sprinting, jumping, throwing, and striking [10,11]. However, few studies have combined squat exercises with flywheel inertia resistance devices. Chiu and Salem [9] compared lower extremity joint kinetics for squat exercises performed using free weights versus a RID, while de Hoyo et al. [3] analysed the effect of an eccentric-overload training program using a YoYo^®^ device. To our knowledge, however, no study has compared force outputs in concentric and eccentric phases with a RID during squatting motions under different loads.

Resistance exercises in unstable conditions are also becoming more popular, and instability training has been implemented by therapists and coaches for rehabilitation and training. Unstable conditions can be achieved with body mass or external loads (e. g., dumbbells or barbells) as the resistance, or by adding unstable surfaces (Swiss, BOSU^®^ balls or natural surfaces such as sand), by using suspended chains, ropes, bands, and by reducing the number of contact points or bases of support. Unstable environments may cause disruption and may lead to postural and joint instability, due to inaccurate neurological adaptation to the environment. Exercises under unstable conditions (UC) increase neuromuscular stress [12,13]. It is generally agreed that force output achieved in different gravitational resistance exercises is lower on unstable surfaces. However, several studies reporting muscle activity during unstable exercises [12,14,15] used absolute rather than relative loads; this procedure may not have allowed a methodologically accurate comparison, because resistance training is usually prescribed as a repetition maximum (RM) load or as a percentage of the 1-RM [16–18]. The use of RID avoids this methodological limitation when exercises are performed at maximum effort under stable conditions (SC) and under UC.

Despite the increasing popularity of flywheel inertia devices and instability for resistance training, no studies comparing force outputs during squats using a RID under both SC and UC are currently available. The aim of the present study was to compare force outputs during the squat exercise using a RID under SC versus UC with different loads and between concentric and eccentric phases. We hypothesized that: 1) the use of the RID would produce higher force outputs under SC than under UC at all loads, 2) force outputs performing squats on a RID would be greatest when the highest moment of inertia of the flywheel and the shortest radius in the cone were selected on both SC and UC, and 3) concentric force output while performing squats on a RID would be higher than eccentric force output for each load under both SC and UC.

## Materials and Methods

**Fig 1:**
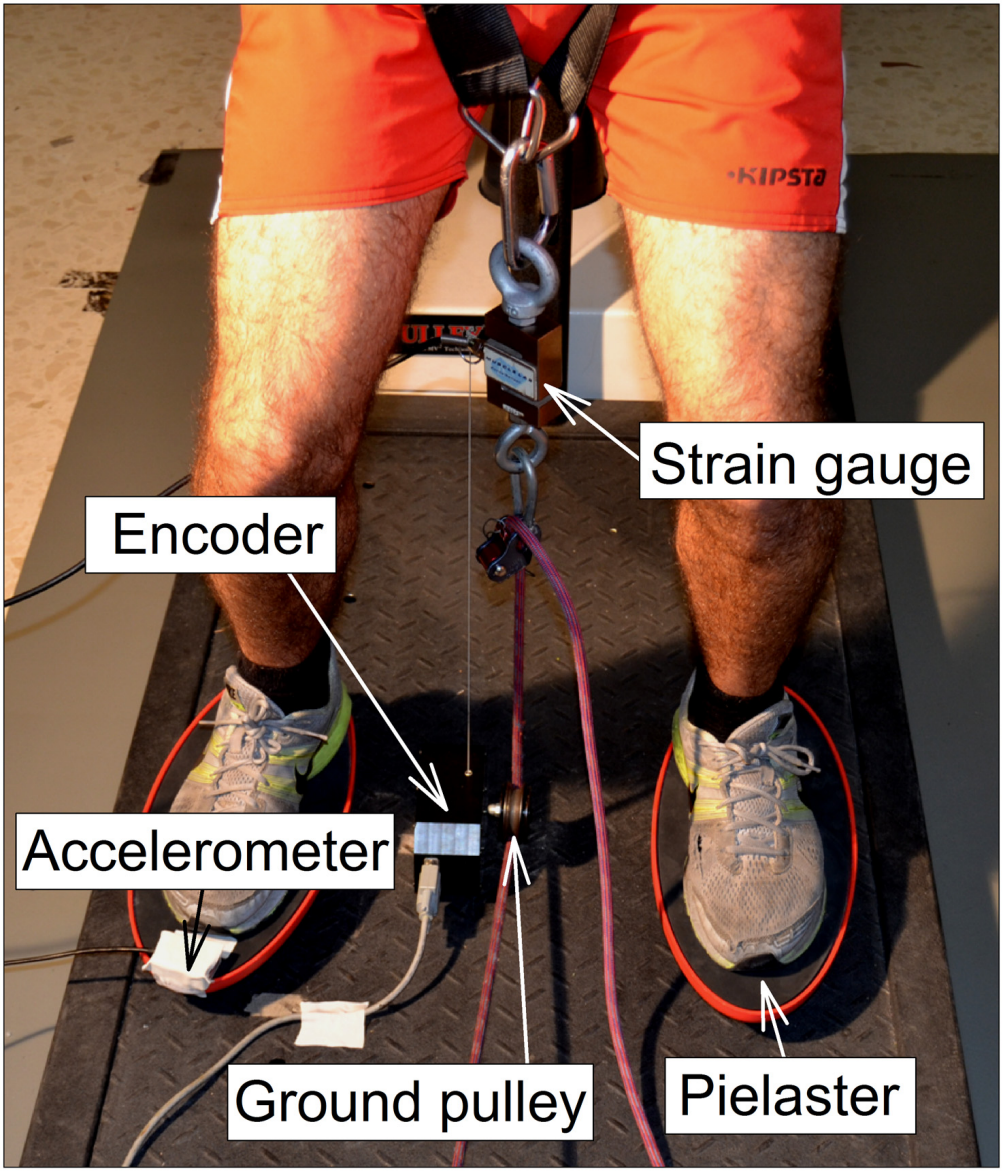
Squat performed on a rotational inertial device under stable conditions.

This study evaluates force outputs when performing squats under stable and unstable conditions with four loads. Participants performed four sets of three repetitions of squats in a random order on a stable platform and on two Pielasters (Biolaster, S. L., Guipúzcoa, Spain). The Pielaster (Fig 1) is an unstable platform consisting of two independent rigid elliptical spheroid platforms; it was chosen because it allows the placement of a pulley on the ground between the independent spheroid platforms. During the exercise, the participant was instructed to apply maximum effort during the concentric phase in each repetition. Data were analyzed to compare force outputs during the squat exercise using a RID under both conditions with different loads, and between concentric and eccentric phases.

### Participants

Thirteen male national-level sprinters volunteered to participate. They each received a clear explanation of the study, including the risks and benefits of participation, and provided written consent prior to inclusion. Their mean ± standard deviation age, height, body mass and, 1-RM were 23.7 ± 3.0 years, 1.80 ± 0.08 m, 77.4 ± 7.9 kg, and 201.9 ± 25.3 kg respectively. They had at least five years of strength-training experience using free-weight squats, but no experience with either RID or instability resistance exercises. The institutional review board for human research (the clinical research ethical committee of the Catalan government’s sports service) approved all the experimental procedures.

### Measurement and instrumentation

#### Rotational inertia device

**Fig 2:**
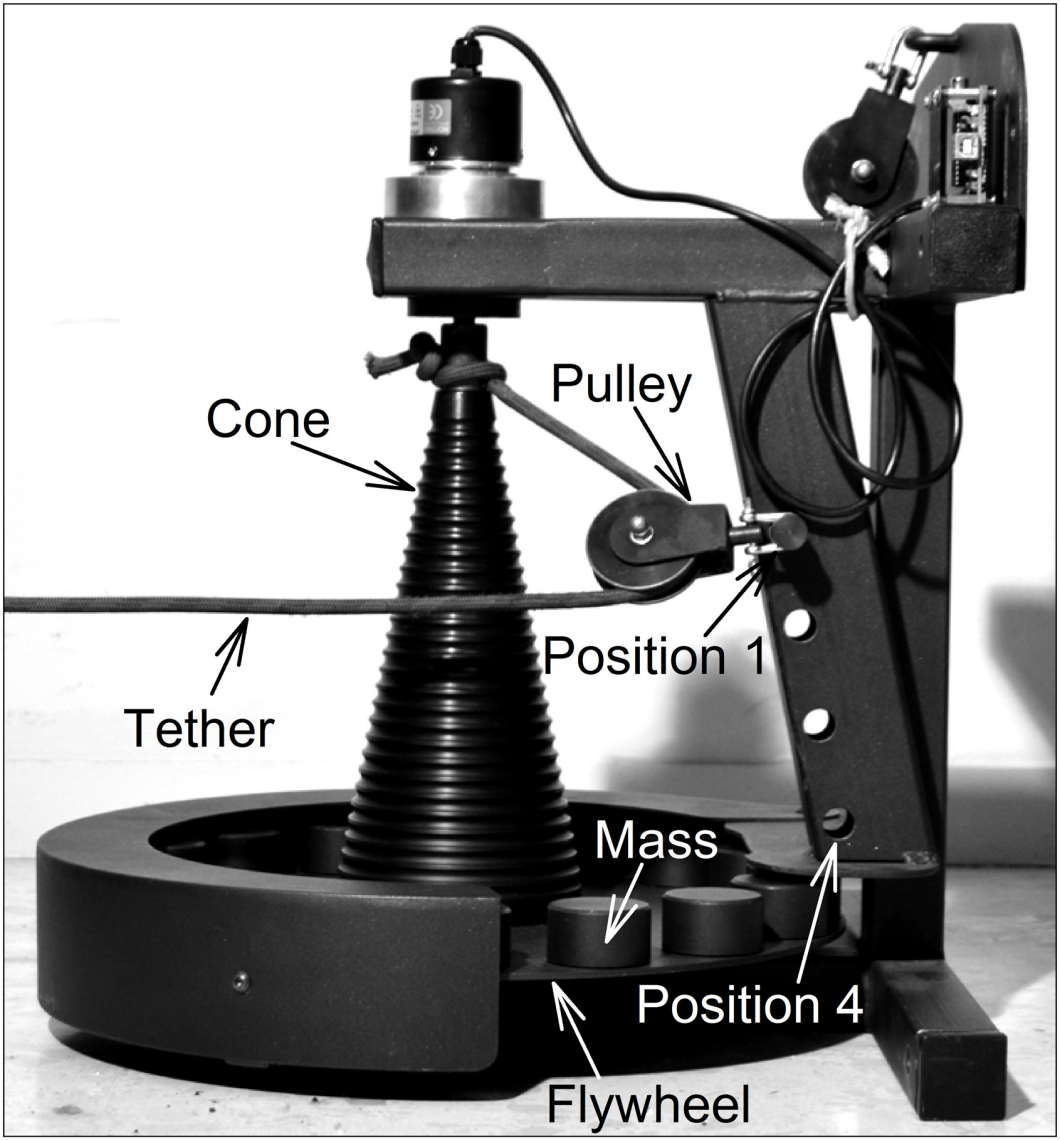
Rotational inertia device. The tether winds (the eccentric phase) and unwinds (the concentric phase) around the cone.

The RID (Byomedic System SCP, Barcelona, Spain) consists of a metal flywheel (diameter: 0.42 m) with up to 16 masses (0.421 kg and 0.057 m diameter each one) which can be added along the top edge of the flywheel perimeter to adjust the overall moment of inertia. The flywheel provides a rotational inertia resistance during coupled concentric and eccentric actions. A fixed axis is located at the center of the beam around which the masses rotate. A cone is attached above the flywheel, and as they spin together a tether winds and unwinds around the cone (Fig 2). The moments of inertia for the RID were 0.12 kg∙m^2^ and 0.27 kg∙m^2^ for 4 and 16 masses respectively.

The length of the tether increases as it unwinds (the concentric phase), but when it has completely unwound, the cone continues to spin, so that the tether then begins to wind around the cone (the eccentric phase). To change resistance to movement it is possible to modify the moment of inertia by adding any number of the 16 masses on the edge of the flywheel and also by selecting four positions (P1, P2, P3 or P4), changing the location of the pulley that is closest to the cone (Fig 2). The greatest force outputs are produced in the upper position (P1), where the rope winds around the narrowest radius of the cone (the lower arm lever). By contrast, the lower position (P4) (the higher arm lever), where a wider part of the cone is used to spin the rope, achieves the highest velocities with the lowest force output. The radius at P1 and P4 were 0.035 m and 0.055 m respectively. Thus, the moment of inertia of the flywheel (4 or 16 masses) combined with the position (1 or 4) of the cone produces four different loads P1-16 (L1), P1-4 (L2), P4-16 (L3) and P4-4 (L4).

#### Force output assessments

Force output was measured using a strain gauge, with a linear encoder (with a time resolution of 10 ms and a spatial resolution of 0.075 mm) used to measure the vertical displacement of the participant performing the squat. Both the strain gauge and the encoder were connected to a MuscleLab 4000e unit (MuscleLab, Ergotest Technology AS, Langesund, Norway). These data were sampled at a frequency of 100 Hz, recorded by the unit and stored on a laptop computer equipped with a data analysis software program (MuscleLab V8.27). The software displays the force, the time course of displacement and the velocity. The strain gauge and the cord of the linear encoder were attached to the harness using carabiners, and the encoder was positioned between the feet, close to the floor pulley (Fig 1). The participant performed three submaximal repetitions in which the velocity increased progressively until near maximum effort was achieved in the third repetition. Then, participants performed one set of three repetitions of squats on the RID at maximum concentric effort. These three last repetitions were computed to calculate mean and peak force. Total force outputs exerted by the participant were calculated as shown in the following equation:

Fp=Fg+m ⋅(a+g) (1)

Where *Fp* is the total force production exerted by the participant which includes the force measured on the gauge (*Fg*), and the force produced against the floor (*m*·*a + m*·*g*), where *m* is the mass of the participant, *a* the acceleration of the participant squatting and *g* is the gravity acceleration. The acceleration in Eq 1 comes from double numerical differentiation of the linear displacement measured by the encoder.

Mean and peak force output measurements for all loads exhibited very high reproducibility: ICC ≥ 0.97 under SC and ICC ≥ 0.96 under UC. The coefficients of variation were ≤ 6.8% and ≤ 12.7% for mean and peak force respectively. This device has been widely used to evaluate dynamic muscle work, and good reliability scores have been reported [19].

### Procedures

Prior to the experiment, participants underwent a familiarization session in which the squat with the RID under both SC and UC was explained, and trial sets were performed at submaximal levels. Instructors emphasized the need for correct exercise technique an stressed the importance of achieving a knee angle of 90° during squats.

The experimental protocol began with a standardized warm-up, after which the participants were randomized to perform one set of three repetitions of squats on the RID with different loads (L1, L2, L3, and L4) under both SC and UC using maximal effort in the concentric phase. A stable platform, specifically designed to maintain the feet at the same height as under UC, was used while performing squats under SC. A rest interval of 2 min was allowed between sets, and the results of the three repetitions of each set were recorded for analysis.

Squats with the RID required participants to wear an adjustable harness equipped with a carabiner. The participant placed his feet at hip width on either side of the pulley located on the ground. This position was marked on the floor and was maintained across sets. The RID tether was then tied to the harness through the strain gauge using carabiners (Fig 1). Finally, the tension of the tether was adjusted while maintaining both legs in extension. Rotation was initiated by winding the tether until reaching 90° of knee flexion, determined by visual inspection. Thereafter, the subject initiated movement, progressively increasing velocity until the third repetition, at which point maximal velocity was reached. Each repetition involved squatting at knee angle of around 90° maintaining the arms against the body under both conditions to prevent counterbalancing. Verbal encouragement was provided to ensure maximal effort and correct technique.

### Statistical Analyses

Data analyses were performed using PASW Statistics for Windows, Version 18.0 (SPSS, Inc., Chicago, IL, USA), and statistical significance was set at *p* < 0.05. Model assumptions were validated by means of the Kolmogorov–Smirnov test of normality and Levene’s test of equality of variances. To assess differences in force and displacement, a four-way repeated measures analysis of variance, considering mass (4 and 16 masses), position (1 and 4), phase (concentric and eccentric) and condition (stable and unstable), as well as their interactions, as fixed factors; the participant was the random factor. All statistically non-significant interactions were removed from the model, and when a statistically significant effect was found, post-hoc comparisons were performed using the Bonferroni correction for multiple comparisons.

## Results

### Force Outputs

**Fig 3:**
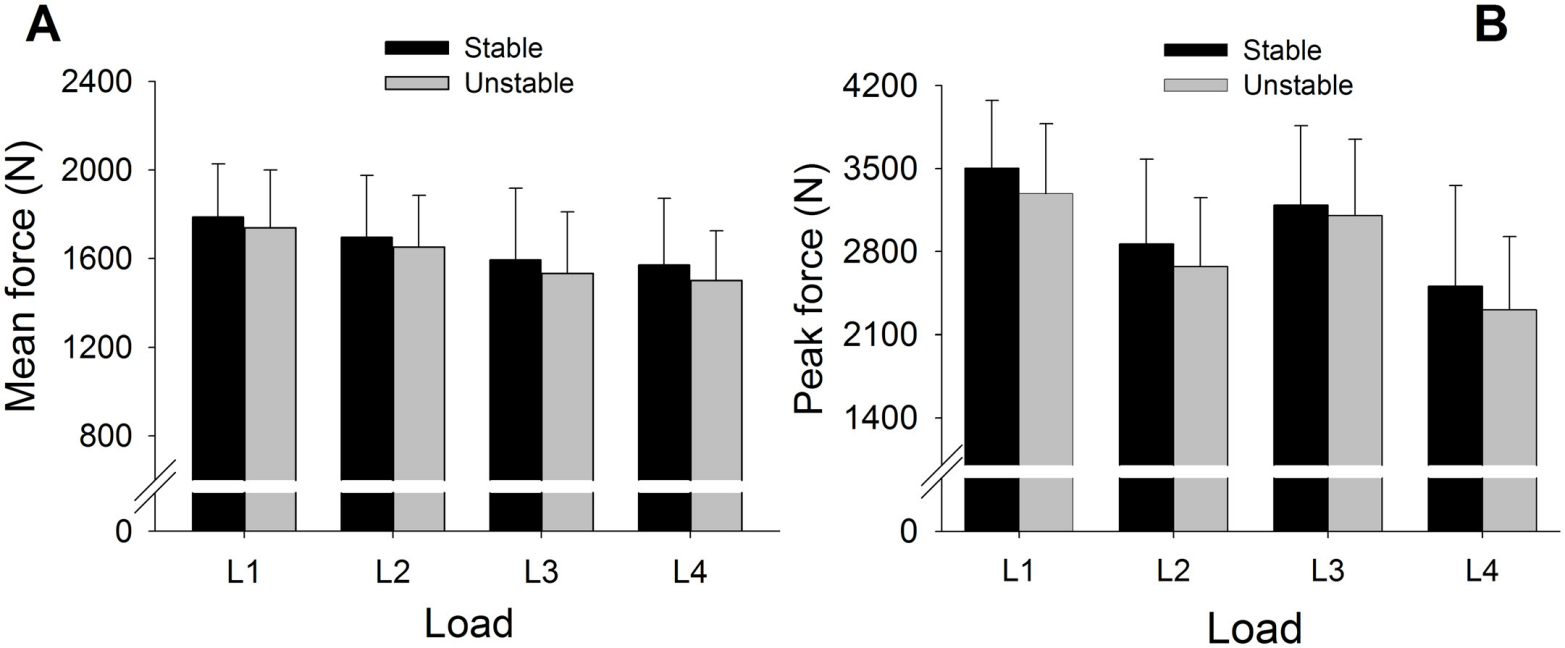
Mean (SD) for mean (A) and peak (B) force outputs under stable and unstable conditions for each load. No statistically significant differences were found between the stable and unstable conditions for each load (n = 13).

No statistically significant differences were found between the SC and UC for any of the loads in terms of mean and peak force (Fig 3) and displacement. Because there were no significant differences between force outputs under SC versus UC for any of the loads, nor any interaction between condition (stable and unstable) and load (weight and position), the data were collapsed. Therefore, the condition was not taken into account in the comparison of force outputs between loads.

**Fig 4:**
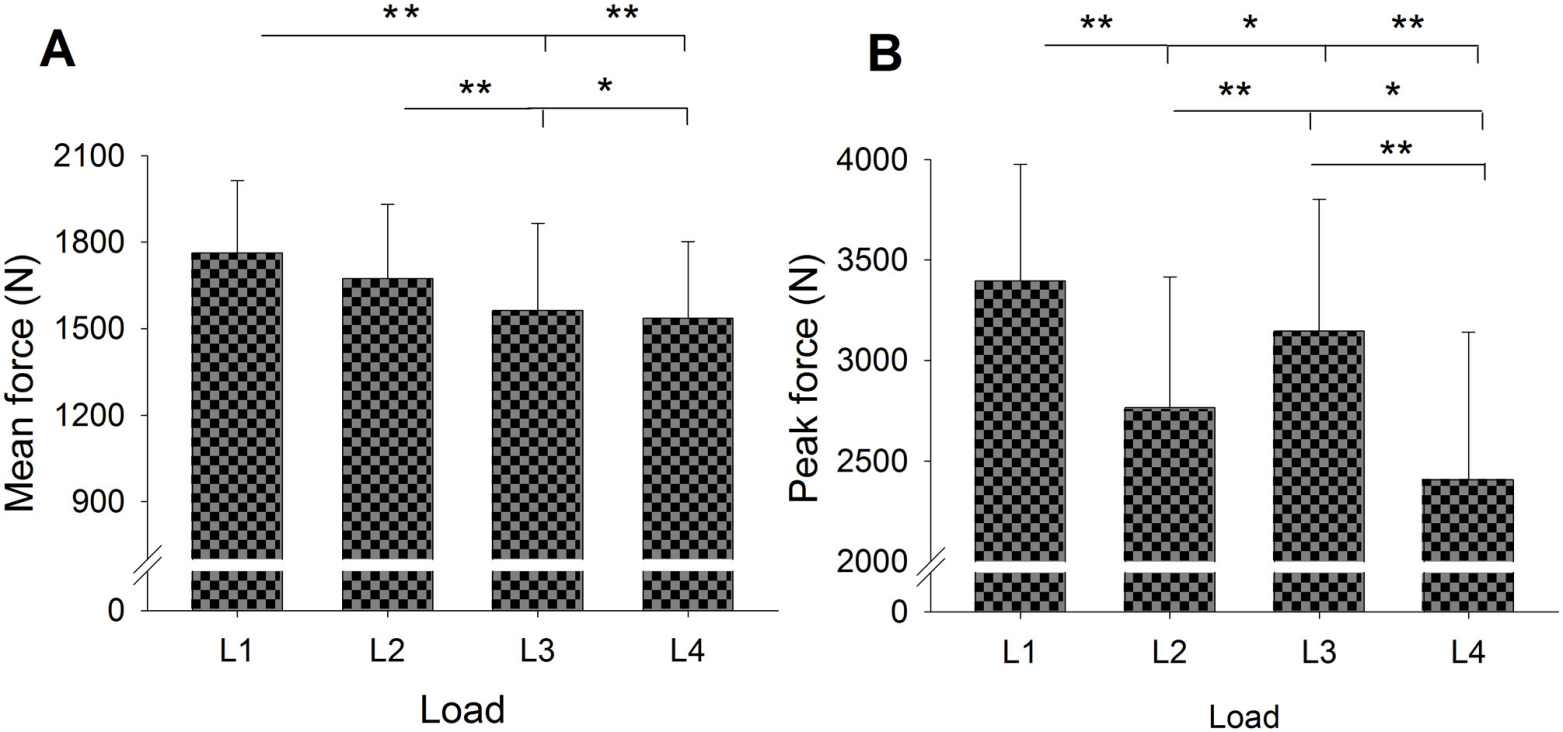
Mean (SD) for collapsed mean force (A) and peak force (B), outputs between loads. (*P < 0.045; **P < 0.001) (n = 13).

Fig 4 shows the collapsed mean and peak force outputs under the four load conditions. A progressive decrease of 13% in force output between loads was observed (L1 > L2 > L3 > L4; 1762.81 ± 251.10 N, 1674.03 ± 257.00 N, 1562.99 ± 301.90 N, 1535.72 ± 265.96 N, respectively). Mean force showed significant differences according to position (Wald Chi-Square = *28*.*029*, *P* < 0.001) and mass (Wald Chi-Square* = 4*.*175*, *P* < 0.041), as noted in Bonferroni post-hoc tests comparing L1, L3 and L4 with L2, L3 and L4 (Wald Chi-Square = *29*.*248*, *P* < 0.001). Lifting different loads resulted in a 29% difference in peak force (L1 > L3 > L2 > L4; 3396.59 ± 589.36 N, 3121.23 ± 654.32 N, 2767.54 ± 656.75 N, 2408.40 ± 745.54 N respectively). Peak force showed significant differences according to position (Wald Chi-Square* = 16*.*149*, *P* < 0.001) and mass (Wald Chi-Square* = 78*.*458*, *P* < 0.001). Post-hoc analyses revealed significant differences for all loads (Wald Chi-Square* = 89*.*119 P* < 0.05). Results for the relative mean outputs ranged from 2.5 times the body mass for the highest load (L1), to 2.4 times for L2, 2.23 for L3, and 2.2 for the lowest load (L4).

### Force Outputs by Phase

**Fig 5:**
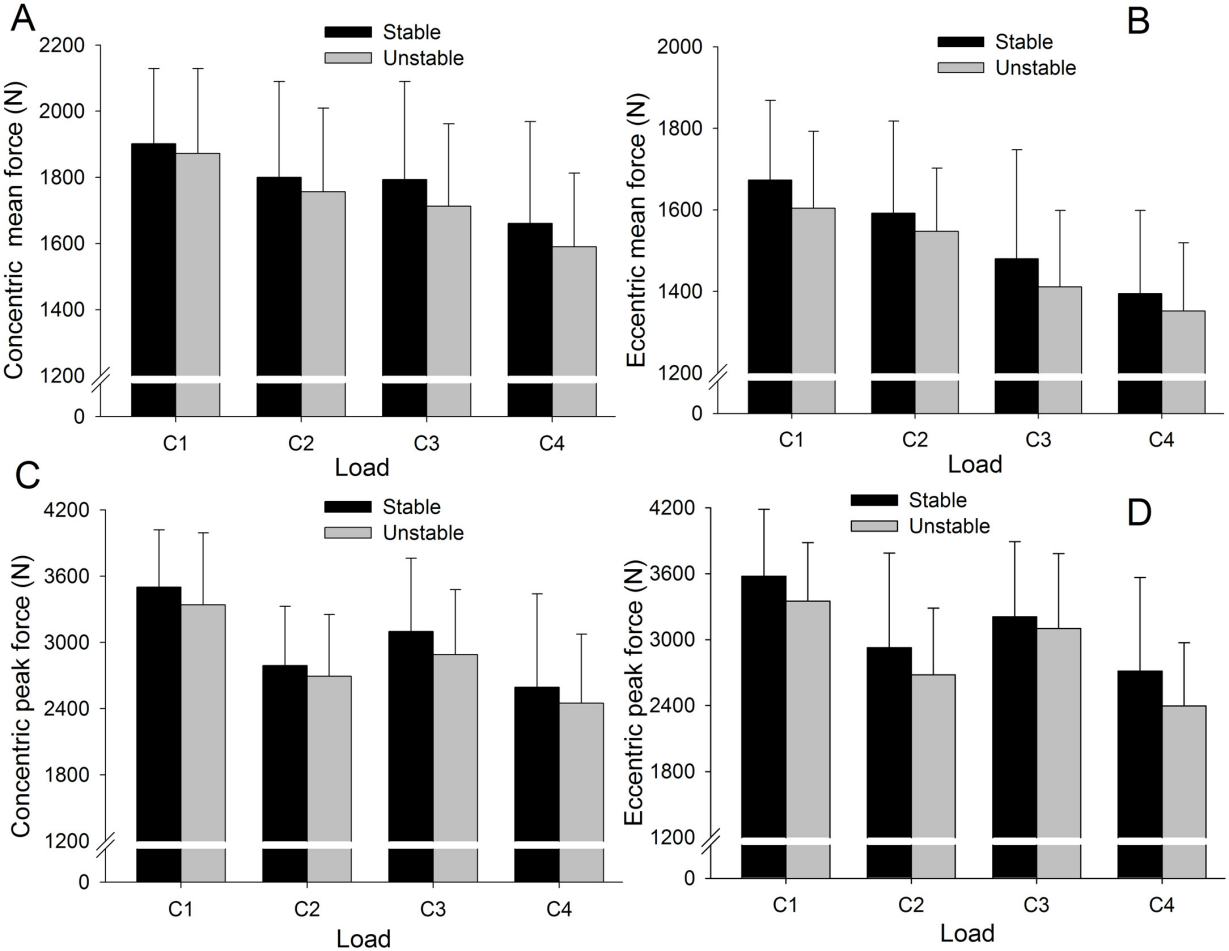
Mean (SD) for concentric mean force (A) and eccentric mean force (B) and concentric peak force (C) and eccentric peak force (D) outputs under stable and unstable conditions for each load. No statistically significant differences were found between the stable and unstable conditions for either phase or any load (n = 13).

No significant differences were found in mean and peak force outputs comparing stable and unstable conditions and concentric and eccentric phases for any of the loads (Fig 5).

**Fig 6:**
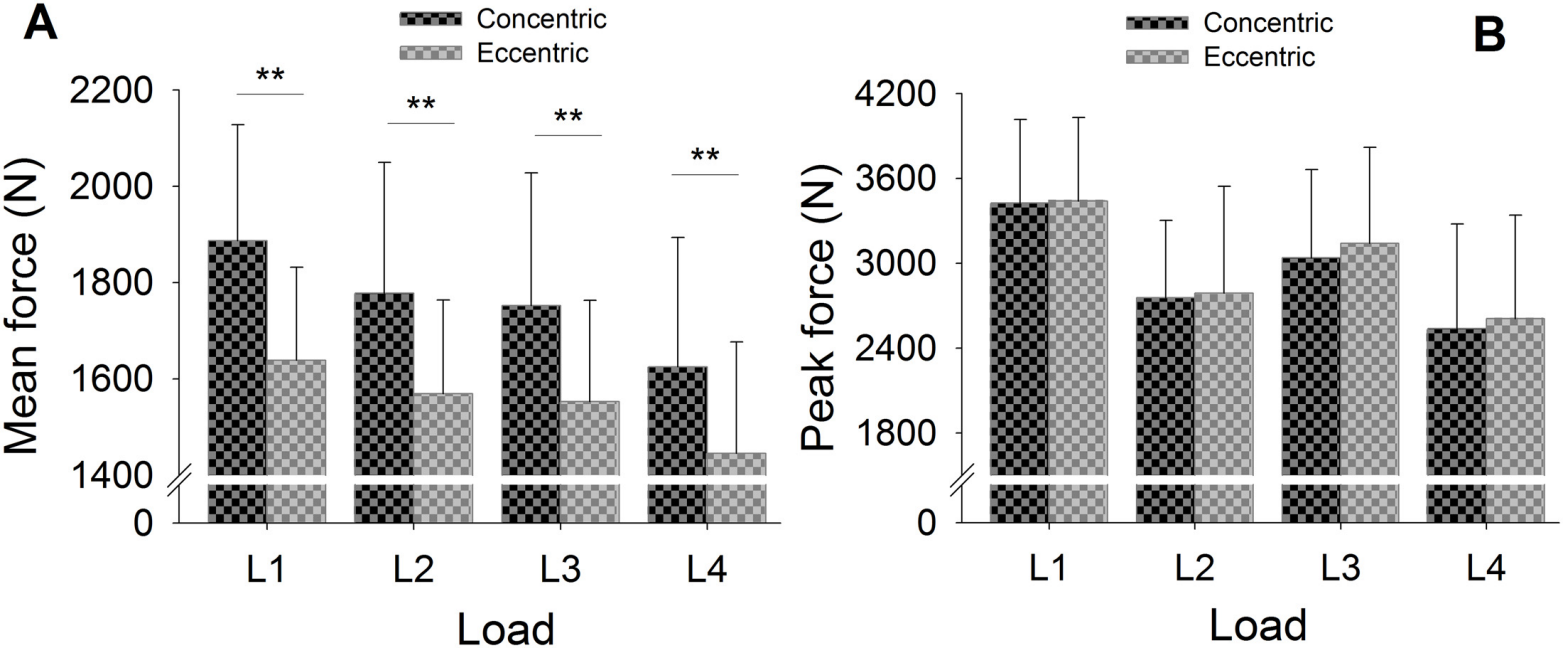
Mean (SD) for concentric and eccentric mean force (A) and peak force (B), outputs for each load. No statistically significant differences were found between the SC and UC for any load **p < 0.001 (n = 13).

As a result, and since there was no interaction between phase (concentric and eccentric), condition (stable and unstable) or load (weight and position), the data were collapsed. Therefore, condition was not taken into account in the comparison of concentric and eccentric force outputs between loads (Fig 6). Different loads resulted in the current concentric mean force (L1 > L2 > L3 > L4; 1886.97 ± 241.12 N, 1778.49 ± 270.54 N, 1752.71 ± 275.34 N, 1625.48 ± 269.25 N respectively) and eccentric mean force outputs (L1 > L2 > L3 > L4; 1638.66 ± 193.87 N, 1569.56 ± 194.17 N, 1553.28 ± 186.45 N, 1445.96 ± 231.57 N respectively). There were statistically significant differences in mean force between positions and masses according to the phase (Wald Chi-Square* = 23*.*465*, *P* < 0.001). Post-hoc tests revealed significant differences for all loads (*P* < 0.001). There were no significant differences in peak force according to phase at any load.

## Discussion

This study was designed to compare the force outputs using a RID while athletes performed squats under SC and UC with four different loads. Surprisingly, the first hypothesis of the study was not borne out: there was a marked similarity in force output during the squat under both conditions for each of the loads.

This result contrasts with those of previous studies, which reported reductions in force output with increasing instability in different gravity-dependent resistance exercises [12,13,20–25]. This discrepancy may be attributable to the type of muscle action, the degree of instability, and the equipment used. In addition, some studies also present methodological limitations, since they either examined absolute intensity [12,14,15] or failed to show normalized muscle outputs [16–18]. Some studies have examined force and power outputs in trainees performing squats on different unstable platforms, including a Bosu^®^ ball [17,26,27], foam blocks [28], inflatable balls [29] or a power board and balance cone [17]. The instability generated by unstable surfaces such as foam blocks and Bosu^®^ balls during the free-weight squat exercise reduced concentric peak force and peak velocity as well as range of motion [28]. Force outputs also decreased with higher levels of instability during isometric squats [17]. Other authors have examined mean power in the concentric phase of squats during the performance of six sets of eight repetitions at 70% of 1-RM [26] and found significantly lower mean power outputs during a free-weight squat exercise on a Bosu^®^ ball than on a stable surface. Similarly, mean power was lower in the entire concentric phase of the free-weight squat exercise with and without countermovement on a Bosu^®^ ball with different weights [27]. However, that study reported that mean power in the entire concentric phase was compromised more by UC than by SC when lifting loads greater than 60% 1-RM. In the current study, performing squats with the RID under UC was associated with a similar reduction in mean and peak force compared with SC across the four loads.

However, given the lack of previous reports in the literature, it is not possible to compare these data and to contrast the degree of instability produced against other unstable surfaces; therefore, the unstable squats assessed in this study may indicate differences between local and global instability. Although the Pielaster perturbed the system, the tension of the tether, which was attached close to the participant’s center of gravity, may have helped to maintain a similar body equilibrium under UC and SC and thus reduce the difficulty of the task. Therefore, the relatively low biomechanical and neuromuscular challenges imposed on the trunk might increase the stability, which might partially explain the slight reduction in force outputs in UC versus SC. On the other hand, unstable surfaces create pressure and tension around the ankle joint and stimulate the mechanoreceptors, thus generating afferent stimuli and reflexive motor responses which increase joint stability. Surfaces such as the Pielaster maybe ideal for stimulating these mechanoreceptors and may help in the prevention or recovery from a range of joint injuries [30]. To activate the vastus medialis oblique, and to enhance the vastus medialis oblique/vastus lateralis ratio in order to prevent or mitigate knee joint dysfunction (the patellofemoral pain syndrome), highly unstable surfaces should be selected [31].

The second hypothesis of the study was partially borne out. Overall, force outputs performing squats on a RID were greatest when the highest moment of inertia of the flywheel and the shortest radius in the cone were selected, in both SC and UC. Specifically, differences in mean force outputs of up to 13% were found between certain loads (Fig 4A). We stress that different moments of inertia using the same position (P1 or P4) did not generate significant differences in mean force outputs (Fig 4A). However, higher force outputs were produced with the cone in the higher position than in the lower position. Despite the 55% increase in the moments of inertia, we did not find differences when using the same position. Therefore, to achieve differences while maintaining the same position, we might have increased the difference between the moments of inertia by more than 55% using the flywheel without masses. In this regard, the manufacturer might consider increasing either the mass of each of the pieces placed on the edge of the flywheel or the number of pieces.

Ten strength-trained men with at least ten years of experience with the barbell squat achieved mean forces close to 1300 N when performing this squat at 10-RM [6]. These forces were slightly lower than the ones recorded in the present study. In another study, 15 healthy men who had 4.5 years of experience with resistance training and were familiar with the free-weight squat exercise showed mean forces of 742.6 ± 222 N when performing maximal isometric squats [17], figures which were also notably lower than ours. In view of this result, and bearing in mind the differences in participants’ characteristics, we suggest that the RID may generate higher mean forces, similar to those produced in traditional hypertrophic and endurance resistance training. However, the RID may be unable to train maximal force (> 85% 1-RM).

Differences in peak force outputs of up to 29% were found between all loads (Fig 4B). Maximal peak forces were reached with 0.57 Kg∙m^2^. For this reason, it seems reasonable to assume that selecting the highest moment of inertia is more effective than changing the positions (P1 or P4) when seeking to increase peak force. Indeed, previous studies reported lower peak forces than ours: below 1500 N when performing the barbell squat at 10-RM [6], and 2186.95 ± 377.34 N during isometric squats [29].

Finally, the third hypothesis of the study was partially supported. Concentric mean force outputs were greater than eccentric force outputs in SC (14%) and UC (15%). Mean forces were lower in the eccentric phase (Fig 6A), probably due to the RID’s mechanical friction: the friction produced while the tether wound and unwound around the cone converted kinetic energy into heat. Likewise, the two pulleys used also produced mechanical friction. Indeed, some investigators have reported higher concentric mean force in a knee extension exercise performed with a specialized YoYo^®^ flywheel device which used two 2.7-kg flywheels with an inertia momentum of 0.07 kg·m^2^ per flywheel [4]. In that study eccentric peak force also exceeded concentric peak force. Similarly, in our study, eccentric peak force was slightly higher than concentric peak force (Fig 6B) when participants performed squatting with the RID under both conditions for all loads. In contrast, Blatnik et al. [32] showed a 15% higher peak concentric force (3767.1 ± 523.2 N) than eccentric force (3196.2 ± 470.6 N) in professional power lifters performing barbell squats. Thus, use of the RID may obtain higher eccentric peak forces than concentric peak forces, something that cannot be achieved performing traditional barbell squats.

Free-weight resistance training typically provides a constant external load during coupled concentric and eccentric muscle actions in sets of consecutive repetitions until failure. However, skeletal muscle inherently has the capability to produce greater force in the eccentric action than in the concentric action which allows for greater loading during the eccentric action. The ability to overcome the gravitational force of a weight depends on the “sticking point” that occurs during the concentric phase of the movement due to changes in biomechanical levers and muscle length. Thus, free-weight resistance exercises using a constant weight require maximal activation only at the “sticking point” of the very last concentric repetition, which results in the failure to lift a particular weight. In contrast, the moment of the inertia of spinning flywheels generates unlimited resistance throughout the entire range of the concentric phase and allows for brief episodes in which eccentric forces exceed the concentric forces [4,7]. Traditionally, strength training programs have been based on gravity-dependent resistance exercises [33]. However, the efficacy of these methods is limited to concentric actions, with lower activation in the eccentric phase [4,5]. Thus, strength-training strategies to prevent muscle strain injuries should also emphasize the eccentric phase of the movement [34]. Moreover, an eccentric-load program (i. e., half-squat and leg-curl exercises using YOYO^®^ flywheel ergometers) reduced muscle-injury incidence and severity in junior elite soccer players [3]. The levels of eccentric peak force generated in the current study were similar to those produced in the concentric phase in both SC and UC and may potentially serve to prevent quadriceps muscle strains.

Our results show that the use of the RID achieved a similar force output during the squat under both conditions for each of the loads. This suggests that the squat exercise with a RID under UC could be incorporated in resistance training programs. The RID allows generation of different force outputs, especially for peak force. Force outputs performing squats on a RID were higher when the highest moment of inertia of the flywheel and the shortest radius in the cone were selected. Peak force was slightly higher in the eccentric than in the concentric phase, and force outputs were comparable under SC and UC. Therefore, the squat exercise performed with a RID may represent an effective alternative to the squat exercise performed with traditional free-weight resistance and with the YoYo^®^ flywheel device for conditioning and strength training. This exercise may be particularly useful for team sport players who need to improve strength and proprioception, and it may also be included as part of injury prevention programs for muscle lesions and ankle and knee joint injuries.
